# Is there a role of proinflammatory cytokines on degenerin‐mediated cerebrovascular function in preeclampsia?

**DOI:** 10.14814/phy2.15376

**Published:** 2022-07-13

**Authors:** Zoltan Nemeth, Joey P. Granger, Michael J. Ryan, Heather A. Drummond

**Affiliations:** ^1^ Department of Physiology and Biophysics University of Mississippi Medical Center Jackson Mississippi USA; ^2^ Department of Pharmacology, Physiology and Neuroscience University of South Carolina School of Medicine Columbia South Carolina USA; ^3^ Institute of Translational Medicine Faculty of Medicine, Semmelweis University Budapest Hungary; ^4^ Department of Morphology and Physiology Faculty of Health Sciences, Semmelweis University Budapest Hungary

**Keywords:** autoregulation, cerebral blood flow, myogenic tone, TNF‐α, βENaC

## Abstract

Preeclampsia (PE) is associated with adverse cerebrovascular effects during and following parturition including stroke, small vessel disease, and vascular dementia. A potential contributing factor to the cerebrovascular dysfunction is the loss of cerebral blood flow (CBF) autoregulation. Autoregulation is the maintenance of CBF to meet local demands with changes in perfusion pressure. When perfusion pressure rises, vasoconstriction of cerebral arteries and arterioles maintains flow and prevents the transfer of higher systemic pressure to downstream microvasculature. In the face of concurrent hypertension, loss of autoregulatory control exposes small delicate microvessels to injury from elevated systemic blood pressure. While placental ischemia is considered the initiating event in the preeclamptic cascade, the factor(s) mediating cerebrovascular dysfunction are poorly understood. Elevated plasma proinflammatory cytokines, such as tumor necrosis factor α (TNF‐α) and interleukin‐17 (IL‐17), are potential mediators of autoregulatory loss. Impaired CBF responses to increases in systemic pressure are attributed to the impaired pressure‐induced (myogenic) constriction of small cerebral arteries and arterioles in PE. Myogenic vasoconstriction is initiated by pressure‐induced vascular smooth muscle cell (VSMC) stretch. Recent studies from our laboratory group indicate that proinflammatory cytokines impair the myogenic mechanism of CBF autoregulation via inhibition of vascular degenerin proteins, putative mediators of myogenic constriction in VSMCs. This brief review links studies showing the effect of proinflammatory cytokines on degenerin expression and CBF autoregulation to the pathological cerebral consequences of preeclampsia.

## Graphical abstract

This brief review summarises studies showing the effect of proinflammatory cytokines on vascular degenerin expression and cerebral blood flow autoregulation to the pathological cerebral consequences of preeclampsia. We propose a mechanism by which cytokines may inhibit one autoregulatory mechanism, referred to as myogenic, or pressure induced, vasoconstriction, which is a common mechanism underlying the cerebrovascular disorders of preeclampsia.

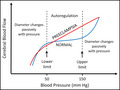

## 1 INTRODUCTION

Preeclampsia (PE), a hypertensive disorder of pregnancy, is one of the most frequent causes of maternal and fetal morbidity and mortality, and affects approximately 2%–6% of pregnancies in the United States and up to 14% of pregnancies worldwide (Force et al., 2017; Lim & Steinberg, 2018; Say et al., 2014; Turbeville & Sasser, 2020). While PE involves multiple organs, the maternal cerebral circulation is particularly susceptible, and consequences of PE include vasospasms, hemorrhagic and ischemic stroke, small vessel disease, and vascular dementia (Miller, 2019). In fact, cerebrovascular disease is the leading cause of maternal mortality in PE, predominantly due to intracerebral hemorrhage or stroke (Duley, 2009; Fishel Bartal & Sibai, 2020). Several factors contribute to the increased susceptibility to cerebrovascular dysfunction including hypertension, inflammation, and reductions in cerebrovascular autoregulatory control of cerebral blood flow (CBF) (Cipolla, 2013; Duncan, Younes, et al., 2020; Warrington et al., 2015; Younes & Ryan, 2019). Both women with PE and a rodent model of placental ischemia used in our laboratory group have elevated CBF out of proportion to rises in blood pressure, which exposes small blood vessels to higher perfusion pressures leading to vascular injury (Granger et al., 2006; Warrington et al., 2019; Zeeman et al., 2004; Zunker et al., 2000). This review will address the evidence supporting a role for placental ischemia‐induced release of proinflammatory cytokines on the loss of CBF autoregulation and propose a mechanism by which cytokines may inhibit one autoregulatory mechanism, referred to as myogenic, or pressure‐induced vasoconstriction.

## 2 PATHOMECHANISMS OF PREECLAMPSIA

The ischemic placenta is thought to set the stage for the initiation of pathophysiological changes in PE (Gilbert et al., 2008; Granger et al., 2001). In normal pregnancy, cytotrophoblasts migrate into the maternal placental spiral arteries, remodeling them from resistance vessels to capacitance vessels capable of handling high fetal metabolic demand. However, in PE, placental spiral arteries fail to properly remodel resulting in poor placental perfusion and exacerbation of placental hypoxia/ischemia (Hecht et al., 2016). In response to ischemia, the placenta releases a mixture of factors, including proinflammatory cytokines [i.e., tumor necrosis factor‐alpha (TNF‐α) and interleukin 17 (IL‐17)], anti‐angiogenic factors [i.e., fms‐like tyrosine kinase 1 (sFlt‐1) and soluble endoglin], and reactive oxygen species (ROS) into the maternal circulation causing widespread endothelial dysfunction that results in enhanced peripheral vasoconstriction and hypertension (Aouache et al., 2018; Goulopoulou & Davidge, 2015; Sargent et al., 2006; Wang et al., 2009). The resulting vasoconstriction and hypertension underlie the proteinuria, cerebrovascular disorders, and brain edema common in PE (Amaral et al., 2015; Gilbert et al., 2008).

Cerebrovascular disorders are the leading cause of maternal mortality in PE, predominantly as a consequence of intracerebral hemorrhage or stroke (Bushnell et al., 2014; Duley, 2009; Fishel Bartal & Sibai, 2020; Miller, 2019). According to a recent cohort study based on the data of Framingham Heart Study, women with PE have a more than threefold higher risk for future stroke (de Havenon et al., 2021). Cerebrovascular disorders of PE include increased blood‐brain barrier (BBB) permeability, subsequent brain edema, visual disturbances, seizures (eclampsia), neurovascular dysfunction, perivascular microhemorrhage, microinfarcts, arteriolar vasculopathy, and stroke (Hammer & Cipolla, 2015; Kontos et al., 1978; Kontos et al., 1981; Logue et al., 2016; Mahendra et al., 2021; Miller, 2019; Paulson, 2002; Ryan et al., 2011; Warrington et al., 2014; Younes & Ryan, 2019; Zunker et al., 2000). A common mechanism underlying the cerebrovascular disorders of PE may be the consequence of CBF dysautoregulation.

## 3 ANIMAL MODELS OF PREECLAMPSIA

Preclinical models of PE are helpful in addressing potential mechanisms underlying preeclampsia. With few exceptions, most rodent models do not develop PE spontaneously but require an intervention before or during pregnancy. Several genetic models are also available, however, these models are hypertensive prior to pregnancy and do not represent “new onset” hypertension. Some of these models of PE are reviewed elsewhere (Bakrania et al., 2022; Gatford et al., 2020; Li et al., 2012).

**FIGURE 1:**
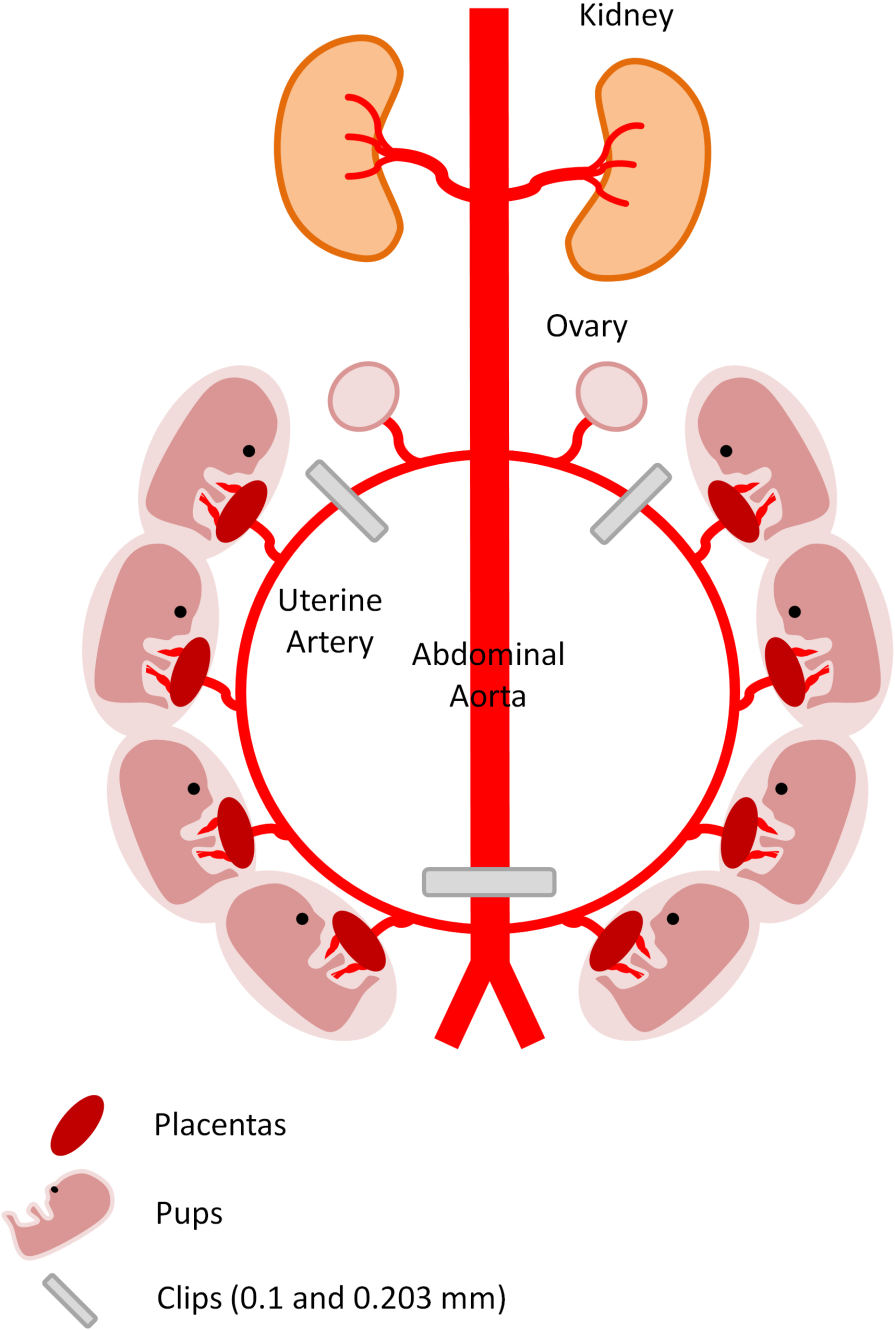
Induction of reduced uterine perfusion pressure (RUPP) in pregnant rats. Laparotomy is performed through an abdominal incision on day 14 of gestation. A silver clip with a 0.203 mm internal diameter is placed around the abdominal aorta right above the iliac bifurcation, and silver clips with 0.1 mm internal diameter were placed around the left and right uterine arcade at the ovarian artery before the first segmental artery. Utero‐placental flow is reduced by ~40% that leads to placental ischemia/hypoxia.

Our laboratory group developed a rodent model that uses a surgical intervention in the last trimester of pregnancy to induce PE (Crews et al., 2000; Granger et al., 2006). In this model, a reduction in placental blood flow is induced by surgical reduction in uterine perfusion pressure (RUPP) generated by clipping the ovarian arteries distal to the ovaries and the lower abdominal aorta (Figure 1). The RUPP model was developed because early studies suggested that the placental spiral arteries failed to remodel from resistance into capacitance‐like vessels in patients with PE, suggestive of reduced blood flow to the placenta and growing fetus. The partial placental ischemia leads to an elevated ratio of placental to fetal mass, a crude measure of “placental insufficiency”, increased expression of placental hypoxic factors [e.g. ROS and hypoxia‐inducible factor 1α, (HIF‐1α)], as well as 40% reduction in utero‐placental flow and increased uterine artery resistive index (Li et al., 2012; Tam Tam et al., 2011; Travis et al., 2021).

The RUPP model also mimics the characteristic features of PE, including hypertension, proteinuria, impaired renal function, increased vascular reactivity, fetal intrauterine growth restriction, and chronic immune activation (Crews et al., 2000; Li et al., 2012). Recent evidence suggests that the RUPP model also mimics cerebral vascular consequences associated with human PE including impaired CBF autoregulation, increased BBB permeability, and brain edema (Ryan et al., 2011; Warrington et al., 2019; Younes & Ryan, 2019). Moreover, RUPP animals have an increased sensitivity to seizure induction, suggesting susceptibility toward seizures observed when PE progresses to eclampsia (Huang et al., 2014; Warrington, 2015). This review will address how elevated proinflammatory cytokines might contribute to the disruption of CBF autoregulation. Much of this research is still ongoing and select concepts from non‐cerebral tissues are extrapolated to cerebral circulation. Thus, this manuscript represents a developing hypothesis.

## 4 MECHANISMS OF CBF AUTOREGULATION

**FIGURE 2:**
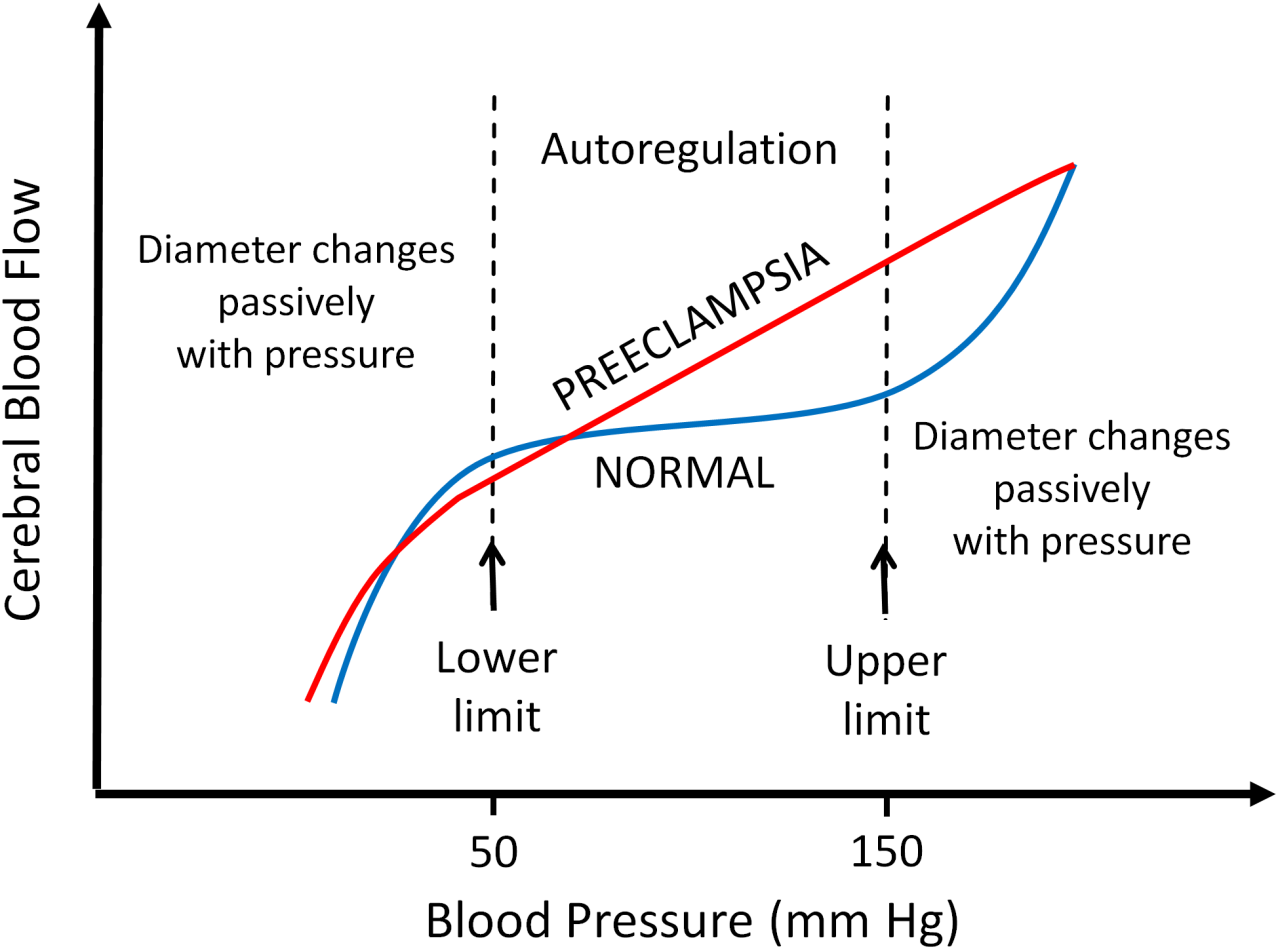
Predicted CBF autoregulatory curves under normal (blue) and preeclamptic/placental ischemic (red) conditions. Under normal conditions, CBF remains relatively stable or “autoregulated” between mean arterial pressures 50–150 mm Hg. In preeclampsia, CBF increases linearly with intraluminal pressure, due to loss of myogenic vasoconstriction of the cerebral vessels. Below 50 mm Hg, small arteries and arterioles fail to actively dilate. At 150 mm hg, small arteries and arterioles are maximally vasoconstricted and further increases in pressure cause dilation.

Autoregulation describes innate vasomotor responses of the small arteries and arterioles in nearly all organs to maintain a near‐constant local blood flow despite changes in perfusion pressure (Claassen et al., 2021). The response, shown in Figure 2, helps to maintain a near‐constant blood flow within a range of cerebral perfusion pressures (~50–150 mm Hg) (Brady et al., 2012; Phillips & Whisnant, 1992). The autoregulatory response is advantageous: it prevents over‐perfusion of local tissue in the face of higher‐than‐normal perfusion pressure and under‐perfusion in the face of lower‐than‐normal perfusion pressure. Autoregulation of flow is accomplished by two primary mechanisms: metabolic and myogenic mechanisms. While these are not the only mechanisms regulating CBF, they are mechanisms shared with other organs such as striated muscle (skeletal and heart), mesentery, and kidney.

The metabolic mechanism is determined by the metabolic demand of the tissues. When perfusion pressure becomes too great, local flow provides excess O_2_ and nutrients, and washes out vasodilatory metabolites (H^+^, CO_2_, adenosine, and lactic acid) from metabolically active tissues, inducing vasoconstriction due to loss of vasodilatory stimuli. When perfusion pressure drops, local flow decreases and vasodilatory factors accumulate leading to vasodilation. The myogenic mechanism controls flow by adjusting vascular tone to changes in perfusion pressure. Increases in perfusion pressure elicit vasoconstriction that counteracts increases in local flow (Claassen et al., 2021). Neurogenic and endothelial factors also contribute to matching cerebral blood flow with changing local demands. Local neural activity is accompanied by the release of vasodilatory mediators from neurons and astrocytes leading to vasodilation and increases in local tissue perfusion. This response is called functional hyperemia or neurovascular coupling (NVC) (Nippert et al., 2018). The cerebral endothelium releases several vasodilatory [e.g. nitric oxide (NO) and endothelial‐derived hyperpolarizing factor (EDHF)] and vasoconstrictor [e.g. endothelins and 20‐hydroxyeicosatetraenoic acid (20‐HETE)] mediators in response to several stimuli (e.g. shear stress and metabolic factors), which regulate vascular tone and therefore CBF (Ashby & Mack, 2021; Faraci & Heistad, 1998; Koller & Toth, 2012). Excellent reviews on this topic can be found elsewhere (Ashby & Mack, 2021; Claassen et al., 2021; Nippert et al., 2018).

This current review will address the known physiological importance of the myogenic mechanism and discuss how disruption of the myogenic signaling mechanism may contribute to cerebrovascular dysfunction in PE. While the consequences of disruption of the autoregulation in the kidney are established, there is limited direct evidence addressing its importance in the cerebral circulation.

## 5 MYOGENIC CONTROL OF CBF AUTOREGULATION

The myogenic mechanism of local blood flow autoregulation is inherent to the vascular smooth muscle cell (VSMC) of small cerebral arteries and arterioles (Schubert et al., 2002). The myogenic response is fact acting (200–300 ms) and provides a rapid, near heart beat‐to‐beat, control of vascular resistance (Bidani et al., 2009; Jernigan & Drummond, 2006; Lidington et al., 2018). While the myogenic mechanism plays a role in the acute control of local blood flow, it may serve a more important role in the long‐term protection of the delicate microcirculation from “barotrauma” caused by the transmission of systemic blood pressure in the kidney and the brain (Faraco & Iadecola, 2013; Schiffrin, 2020; Wang et al., 2020). Hypertension exposes microvessels to chronically elevated perfusion pressures. The adaptive response in renal and cerebral vessels is characterized by extracellular matrix expansion, infiltration of inflammatory cells, inward remodeling of small arteries and arterioles and eventually capillary loss (rarefaction) (Faraco & Iadecola, 2013; Schiffrin, 2020; Wang et al., 2020). While the impact of a loss of autoregulation on vascular injury in the kidney has been studied, its impact on the cerebral vasculature is not as clear. However, a few studies link loss of cerebral autoregulation with loss of BBB function, rarefaction, and/or cognitive loss in genetically modified animal models (Fan et al., 2015; Wang et al., 2020). These findings suggest that the myogenic mechanism may be critical to preventing the vascular injury (BBB disruption and brain edema, arterial wall remodeling) of the maternal cerebral circulation from prolonged increased perfusion pressure in hypertension associated with PE.

How is the myogenic response mediated? The mechanism(s) underlying initiation of the myogenic mechanism remains unresolved, although candidates have been proposed. There are several aspects of the myogenic response that are generally agreed upon. First, the response is mechano‐dependent. VSMCs are wrapped circumferentially around small arteries and arterioles and increases in perfusion pressure induce a transient dilation, which stretches or elongates VSMCs. Second, the elongation of the VSMC initiates a “mechano‐electrical” coupling event leading to VSMC membrane depolarization, calcium influx, and VSMC contraction (Franklin et al., 1997; Numaguchi et al., 1999). Third, multiple molecules are required for a “normal” myogenic response, including extracellular matrix (ECM) molecules, integrins, membrane channels, and G‐protein coupled receptors (GPCRs) (Delmas & Coste, 2013; Kellenberger & Schild, 2002; Welsh et al., 2002). What remains unresolved is the precise molecular mechanism(s) that initiates the response. Several candidates have been suggested, including members of the Transient Receptor Potential (TRP) channel coupled to GPCRs, Piezo channels, and degenerin channels. Our laboratory has focused on the role of the evolutionarily conserved family of degenerin proteins, known to act as mechano‐electrical couplers in model organisms, as important transducers of the vascular‐induced stretch signal. The evolutionary evidence favoring a role for degenerin channels as mechano‐electrical couplers in VSMCs is discussed elsewhere (Drummond, 2021).

## 6 THE DEGENERIN PROTEINS: ROLE IN MYOGENIC CONSTRICTION

Degenerin proteins have a strong evolutionary link to mechanosensation in neuronal and muscle tissues of the nematode *Caenorhabditis elegans* and fly *Drosophila melanogaster* (Arnadottir & Chalfie, 2010; Cheng et al., 2010; Drummond et al., 2004; Drummond & Stec, 2015; Gannon et al., 2008; Kim et al., 2013; W. Li et al., 2006; Mano & Driscoll, 1999). The name “degenerin” was coined when early studies described gain‐of‐function mutations in *C. elegans* that induced neuronal swelling and lysis, or degeneration (Chalfie & Wolinsky, 1990; Driscoll & Chalfie, 1991). Subsequent mutations in genes expressed in touch receptor neurons were associated with a lack of behavioral avoidance responses to gentle body touch (Ben‐Shahar, 2011; Garcia‐Anoveros & Corey, 1996; Kellenberger & Schild, 2002). The degenerin family includes two groups that are expressed in mammals: the Epithelial Na^+^ Channels (ENaC) and Acid Sensing Ion Channels (ASIC) (Drummond, Jernigan, & Grifoni, 2008).

ENaC channels are widely known for their role in Na^+^ and water transport in the renal, lung, and gut epithelia. In these tissues, αβγ subunits associate with constitutively active Na^+^ selective channels (Ben‐Shahar, 2011; Drummond, 2012; Drummond et al., 2004; Drummond, Grifoni, & Jernigan, 2008; Drummond & Stec, 2015; Ge et al., 2012; Grifoni et al., 2010; Guan et al., 2009; Jernigan & Drummond, 2005, 2006; Jernigan et al., 2008; Kim et al., 2012). ASIC channels are characterized by their signature gating response to rapid increases in extracellular H^+^ concentration. At least five genes encoding ASIC channels have been identified (ASIC1‐5). Most ASIC channels are predominantly expressed in central and peripheral sensory neurons where they contribute to learning and memory function, fear, and neuronal sensation of touch and pain sensation with inflammation (Abdelhamid & Sluka, 2015; Gu & Lee, 2010; Wemmie et al., 2002; Wemmie et al., 2003; Wemmie et al., 2013).

Our laboratory and other's have shown that certain ENaC and ASIC subunits also play critical roles in mediating renal and cerebral vascular function, namely pressure‐induced constriction and myogenic autoregulation of blood flow (Drummond et al., 2004; Drummond et al., 2011; Gannon et al., 2008; Gannon et al., 2015; Ge et al., 2012; Grifoni et al., 2010; Guan et al., 2009; Jernigan & Drummond, 2005, 2006; Kim et al., 2012; Kim et al., 2013; Nagasawa & Imig, 2013; Nemeth et al., 2021). In the kidney and brain, βENaC, γENaC, and ASIC2 are expressed in VSMCs and required for normal pressure‐induced constrictor responses (Drummond, 2012; Drummond et al., 2004; Drummond, Grifoni, & Jernigan, 2008; Drummond, Jernigan, & Grifoni, 2008; Gannon et al., 2015; Ge et al., 2012; VanLandingham et al., 2009). In the kidney, βENaC and ASIC2 are required for normal myogenic blood flow autoregulation and loss of βENaC or ASIC2 is associated with mild renal injury, presumably due to loss of the “protective” myogenic response (Gannon et al., 2015; Jernigan & Drummond, 2005, 2006). Direct evidence of the role of vascular ENaC on autoregulation of CBF is not available; however, the importance of ENaC/ASIC subunits in myogenic responses in isolated vessels suggests that degenerin‐mediated myogenic constriction also contributes to CBF autoregulation (Faraci et al., 2019; Gannon et al., 2015; Gannon et al., 2008; Grifoni et al., 2010; Nemeth et al., 2021; VanLandingham et al., 2009).

There are four major lines of evidence that support the importance of ENaC proteins to myogenic constriction and blood flow autoregulation. First, in our earliest studies, we used the broad spectrum ENaC inhibitor amiloride and its analog Benzamil in isolated rat middle cerebral artery (MCA) and mouse renal interlobar artery and found that ENaC inhibition abolishes myogenic constriction, but not agonist (depolarization or α‐adrenergic receptor)‐induced constriction (Drummond et al., 2004; Jernigan & Drummond, 2005). Sensitivity of the myogenic constrictor responses to ENaC inhibitors was subsequently confirmed by other investigations (Guan et al., 2009; Kim et al., 2013; Nagasawa & Imig, 2013). Second, to determine the involvement of β and γENaC, we used overexpression of dominant‐negative constructs and small interfering RNA (siRNA) to specifically disrupt their expression in isolated mouse renal interlobar arteries. Both gene silencing approaches abolished myogenic, but not agonist induced, constriction (Jernigan & Drummond, 2006). A subsequent study by Kim et al. confirmed a similar dependence of posterior cerebral artery myogenic responsiveness on β and γENaC (Kim et al., 2013). Third, we have shown that the myogenic response in the MCA is nearly abolished in mice with reduced levels of βENaC or in ASIC2 knockout mice (Gannon et al., 2008; VanLandingham et al., 2009). We have also shown that βENaC and ASIC2 are required for myogenic constriction in renal vessels and autoregulation of whole kidney blood flow (Drummond et al., 2011; Gannon et al., 2015; Ge et al., 2012; Grifoni et al., 2010). Fourth, we showed that overexpression of full‐length βENaC in isolated MCAs in aged female wildtype mice enhances myogenic constriction (Nemeth et al., 2021). While these studies support a critical role for ENaC in myogenic constriction in renal and cerebral vessels, it is important to note that these evidences are limited to myogenic responsiveness of small artery segments and not arterioles, therefore future studies are necessary to establish the role of degenerins in myogenic constriction in parenchymal arterioles, the major site of vascular resistance.

Collectively, downregulation of vascular ENaC proteins may be a potential mechanism mediating the loss of cerebral myogenic vasoconstriction and the myogenic mechanism of blood flow autoregulation associated with PE.

## 7 DYSAUTOREGULATION OF CBF IN PREECLAMPSIA

Pregnancy is associated with maternal hemodynamic changes aimed at supporting the developing fetus. The major hemodynamic changes in normal pregnancy include increased blood volume and cardiac output, and decreased systemic vascular resistance (Cipolla, 2013; Poston et al., 1995). Despite these circulatory changes, CBF autoregulation maintains appropriate during normal pregnancy (Cipolla et al., 2012; Cipolla et al., 2011). In contrast, studies in animal models of placental ischemia and human patients with PE show that CBF autoregulation is impaired, as shown in Figure 2 (Miller, 2019; Riskin‐Mashiah & Belfort, 2005; Riskin‐Mashiah et al., 2001; Ryan et al., 2011; Sonneveld et al., 2014; van Veen et al., 2013; van Veen et al., 2015; Warrington et al., 2019; Warrington et al., 2014; Zeeman et al., 2004; Zunker et al., 1995). Clinical studies measuring the CBF velocities of large cerebral arteries via transcranial Doppler sonography (TCD) or magnetic resonance (MR) imaging reported increased CBF velocities in women with PE compared to normotensive control subjects (Riskin‐Mashiah & Belfort, 2005; Riskin‐Mashiah et al., 2001; Sonneveld et al., 2014; van Veen et al., 2013; van Veen et al., 2015; Zeeman et al., 2004; Zunker et al., 1995). Similar findings were observed in the RUPP rat model of preeclampsia. Studies from our laboratory using laser Doppler flowmetry have shown increased CBF in placental ischemic rats compared to normal pregnant rats (Warrington et al., 2019; Warrington et al., 2014). Importantly, a previous study by our research group showed reduced myogenic constriction of the isolated MCA in placental ischemic rats (Ryan et al., 2011). The impaired myogenic reactivity likely accounts for the impaired CBF autoregulation in this model. The signaling mechanisms underlying the loss of pressure‐induced constriction of cerebral vessels in PE are unclear, however, we hypothesize that immune mechanisms may be responsible.

Several lines of evidence suggest that immune mechanisms contribute to altered CBF autoregulatory responses in preeclampsia. First, circulating proinflammatory cytokines, such as TNF‐α, IL‐1β, IL‐2, IL‐6, IL‐7, IL‐8, IL‐17, and IFN‐γ are elevated in humans with PE and animal models (Conrad et al., 1998; Cornelius, 2018; Freeman et al., 2004; LaMarca et al., 2005; Ma et al., 2019; Spence et al., 2021; Vince et al., 1995). Second, inhibition of select cytokines attenuates the development of hypertension associated with PE (LaMarca et al., 2008; Warrington et al., 2015). A number of studies show that proinflammatory cytokines contribute to the development of hypertension through several mechanisms including (1) maternal vascular endothelial dysfunction, (2) enhanced formation of vasoconstrictors, (3) decreased production of vasodilators, and (4) activation of the sympathetic nervous system (Cornelius, 2018; Granger et al., 2001; Matsubara et al., 2015). A small number of animal studies suggest that infusion of proinflammatory cytokines TNF‐α or IL‐17 during the last trimester of normal pregnancy is sufficient to alter CBF autoregulation (Duncan et al., 2021; Duncan, Younes, et al., 2020; Warrington et al., 2015). TNF‐α infusion into pregnant rats increased brain water content, likely a result of increased BBB permeability (Warrington et al., 2015). A follow‐up study confirmed that the CBF autoregulation was also modestly disrupted in this model, which may have contributed to BBB disruption (Duncan, Younes, et al., 2020). Infusion of IL‐17 during the last trimester of normal pregnancy had a minor impairment of CBF autoregulation at high perfusion pressures (Duncan et al., 2021). IL‐17 elicited subtle changes in the myogenic response in cerebral arteries; maximal myogenic tone was achieved at lower intraluminal pressures, suggesting a reduced buffering capacity at the highest intraluminal pressures. These studies provide evidence that neither TNF‐α nor IL‐17 alone are sufficient to recapitulate the features of impaired cerebral vascular function with placental ischemia and multiple cytokines may act synergistically to disrupt cerebrovascular function. It is likely that other proinflammatory cytokines, such as IL‐6, IL‐1β, IL‐2, and IFN‐γ are also involved in the cerebrovascular dysfunction in PE and their potential role should be elucidated by future studies. Importantly, studies in both women with PE and animal models of placental ischemia show reduced expression of ENaC proteins in the cerebral vasculature and the placenta suggesting a possible link between proinflammatory cytokines and cerebral vascular degenerin proteins (Ryan et al., 2011; Wang et al., 2013).

## 8 COULD PROINFLAMMATORY CYTOKINES DISRUPT MYOGENIC CEREBRAL AUTOREGULATION BY INHIBITING DEGENERIN EXPRESSION?

ENaC expression in epithelial tissues is suppressed by proinflammatory cytokines. Proinflammatory cytokines inhibit the expression of ENaC proteins in epithelial cells in the lung, gut, and kidney leading to loss of Na^+^ and water reabsorption (Magalhaes et al., 2016; Schmidt et al., 2007; Wynne et al., 2017). In acute respiratory syndrome, reduced expression of alveolar ENaC proteins is associated with fluid accumulation in the alveoli (Wynne et al., 2017). Inflammatory bowel disease is associated with the downregulation of apical ENaC proteins in the gut epithelial cells that results in low electrolyte absorption and diarrhea (Magalhaes et al., 2016). In septic kidney injury, the increased Na^+^ excretion in the collecting duct is a consequence of decreased expression of tubular ENaC proteins in response to inflammation (Schmidt et al., 2007). Considering the finding that the myogenic response of small cerebral arteries and myogenic CBF autoregulation are dependent on normal ENaC expression, it follows that proinflammatory cytokines might also inhibit vascular ENaC expression.

**FIGURE 3:**
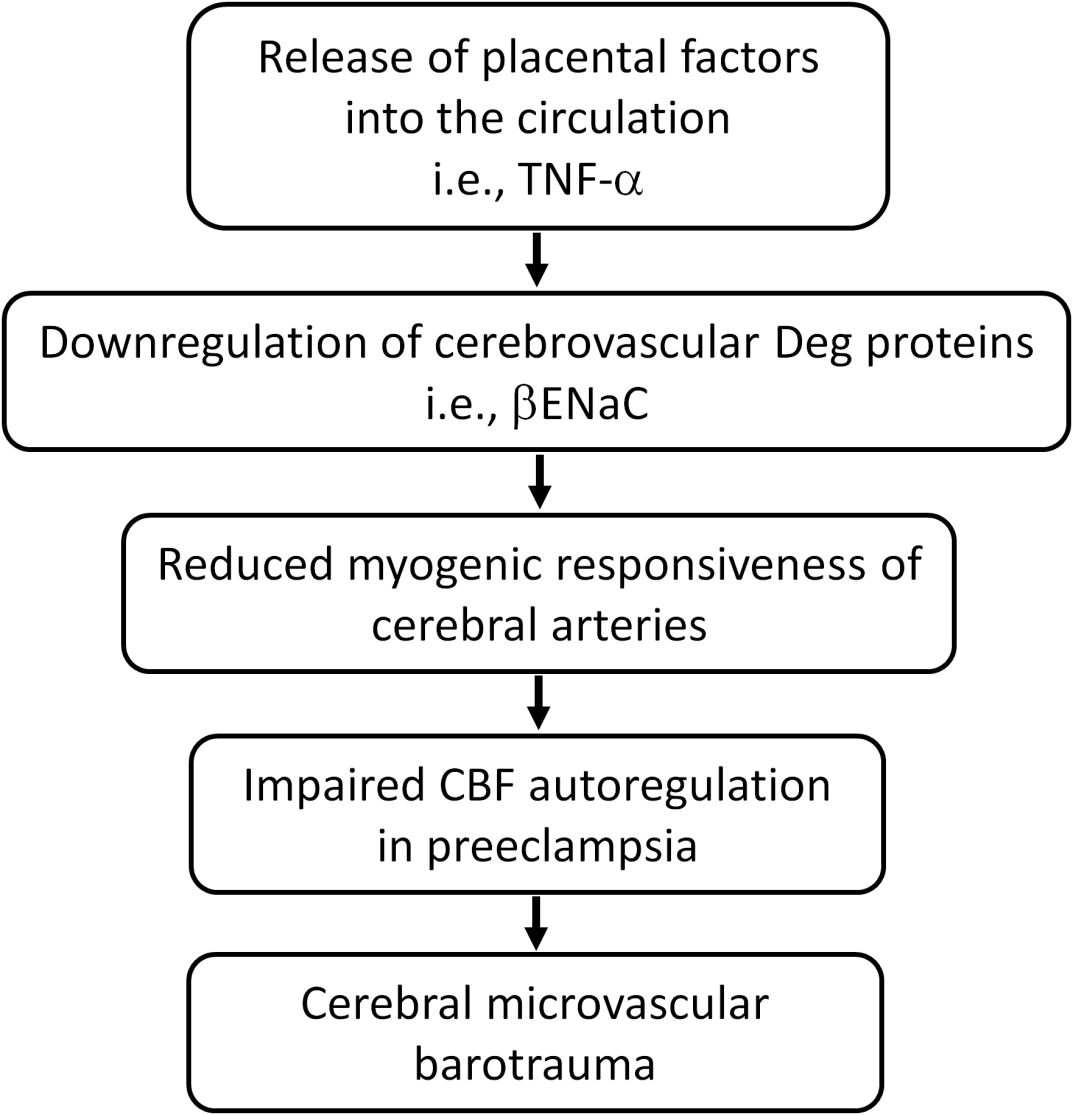
Proposed mechanism by which proinflammatory cytokines disrupt CBF autoregulation in preeclampsia through inhibition of vascular βENaC expression and cerebral myogenic constriction. The ischemic placenta releases placental factors, including proinflammatory cytokines, such as TNF‐α into the circulation, which inhibit the expression of degenerin (Deg) proteins, such as βENaC in the cerebral vessels. Downregulation of cerebrovascular βENaC leads to reduced myogenic responsiveness of small cerebral arteries and arterioles leading to impaired CBF autoregulation in preeclampsia, which ultimately results in cerebral microvascular barotrauma.

There are two lines of evidence that support our hypothesis that proinflammatory cytokine‐dependent inhibition of vascular ENaC contributes to the loss of myogenic CBF autoregulation in preeclampsia. First, there is an inverse relationship between ENaC expression and preeclamptic symptoms. In the rodent model of placental ischemia, expression of βENaC in cerebral vessels is reduced, compared to normal pregnant animals (Ryan et al., 2011). Second, normal rodent pregnancy superimposed with elevated levels of TNF‐α or IL‐17 inhibits βENaC, and possibly ASIC2 expression, in cerebral vessels and isolated VSMCs in vitro (Duncan, Granger, et al., 2020; Duncan et al., 2021; Duncan, Younes, et al., 2020). In vitro studies suggest that these cytokines inhibit βENaC expression in VSMCs via mitogen‐activated protein kinase/c‐jun N‐terminal kinase (MAPK/JNK) pathways (Duncan, Granger, et al., 2020; Duncan, Younes, et al., 2020). These findings are consistent with the hypothesis that elevated circulating inflammatory cytokines inhibit vascular βENaC expression, leading to a loss of myogenic CBF autoregulation (Figure 3). Additional studies demonstrate that (1) blockade of select cytokines prevents PE‐mediated loss of VSMC βENaC, cerebral myogenic vasoconstriction, and autoregulation and (2) rescue of VSMC βENaC in PE protects against loss of autoregulation and cerebrovascular dysfunction (BBB disruption, edema, etc.) are required to establish the hypothesis.

## SUMMARY

Placental ischemia/hypoxia contributes to the development of preeclampsia by inducing an increased release of proinflammatory cytokines from the placenta into the circulation. Proinflammatory cytokines are the key players in the pathophysiology of preeclampsia including the cerebrovascular dysfunctions, which are the leading cause of maternal mortality in preeclampsia. Cerebrovascular dysfunction associated with preeclampsia, such as increased BBB permeability and brain edema is attributed to the reduced ability of cerebral vessels to constrict in response to an increase in perfusion pressure, i.e., impaired myogenic‐mediated CBF autoregulation. Studies from our research group demonstrated that proinflammatory cytokines, TNF‐α, and IL‐17 inhibit (1) βENaC expression in VSMCs in vitro, (2) cerebral vessel βENaC expression in vivo, and (3) myogenic CBF autoregulation (Duncan, Granger, et al., 2020; Duncan, Younes, et al., 2020). Thus, we propose that select proinflammatory cytokines disrupt CBF autoregulation in preeclampsia through inhibition of vascular βENaC expression and cerebral myogenic vasoconstriction (Figure 3), leading to a progression of microvascular injury in the brain. How will this information lead to the development of therapies for preeclampsia? If suppression of VSMC βENaC increases cerebral vascular injury, targeting pathways that inhibit these channels could protect the cerebral vasculature from injury in preeclampsia.

## ETHICS STATEMENT

No humans or animals were used in the present publication, except as used in original cited published articles.
